# The dual burden of a rare cancer: psychosocial impact of disease symptoms and unmet care needs in cutaneous T-cell lymphoma

**DOI:** 10.3389/fpsyg.2026.1786000

**Published:** 2026-06-03

**Authors:** Alina Wacker, Jana Stucke, Rudolf Stadler, Sabine Steinke

**Affiliations:** 1 Medical Faculty OWL, University of Bielefeld, Bielefeld, Germany; 2 University Clinic for Dermatology, Ruhr University of Bochum, Minden, Germany

**Keywords:** cutaneous T-cell lymphoma, health-related quality of life, patient experiences, psychosocial burden, qualitative research, rare diseases, unmet care needs

## Abstract

**Introduction:**

Cutaneous T-cell lymphoma (CTCL) is a rare, chronic cancer with different stages and the risk for systemic involvement. Although quantitative data show that the disease negatively impacts patients’ quality of life (QoL), in-depth qualitative research into patients’ daily lives and care experiences is limited. This study aims to explore patients’ experiences along the full care pathway, (unmet) needs, and health-related quality of life (HRQoL) in Germany, with a special focus on the psychosocial impact of living with this rare cancer.

**Methods:**

In a qualitative study, 19 semi-structured interviews were conducted between October 2024 and February 2025 (median duration 47 min). Patients aged 35 to 80 years (11 female, 8 male) were recruited using purposive sampling through a skin cancer center and two German support groups, representing 9 centers across Germany. Analysis followed Kuckartz’s qualitative content analysis approach.

**Results:**

Most patients experienced HRQoL impairment across physical, psychological and social domains. Common impacts included pruritus and sleep disturbances, social withdrawal due to visible skin changes, stigmatization experiences, and psychological burden related to prognostic concerns and altered self-perception. Quality of care emerged as a factor influencing HRQoL: adequate care was perceived as supportive for coping with the disease and psychosocial well-being, while unmet care needs often created an additional burden. Major care challenges included diagnostic delays, insufficient specialized knowledge, fragmented care, trivialization of the disease and inadequate information provision. Both early- and advanced-stage patients showed HRQoL impairment, but with different patterns: patients in early stages primarily experienced psychosocial burden due to unmet care needs, while advanced-stage patients reported greater physical symptom burden.

**Conclusion:**

Patients with CTCL face a dual psychosocial burden from disease-specific impairments and care challenges due to the disease’s rarity. The latter may be prevented through specialized, coordinated care using person-centered approaches. Understanding patients’ experiences and (unmet) needs is essential for developing supportive care pathways. Positive care experiences reported in this study provide valuable insights for clinical practice. Future research should validate these findings across different healthcare systems and incorporate them into CTCL-specific HRQoL measurement tools.

## 1 Introduction

Cutaneous T-cell lymphomas (CTCLs) are rare subtypes of non-Hodgkin lymphomas, primarily manifesting in the skin (Willemze, 2022). The most prevalent forms are mycosis fungoides (MF) and Sézary syndrome (SS), with MF accounting for approximately 62% and SS for 3% of all CTCL cases (Dobos et al., 2020). The incidence of CTCL ranges from 0.29 to 0.87 per 100,000 person-years internationally (Dobos et al., 2020; Cai et al., 2022).

Early stages (IA-IIA) typically present as patches that may evolve into plaques, while advanced stages (IIB-IV) are characterized by tumorous lesions, ulcerations, and erythroderma, often with systemic involvement (Kempf and Mitteldorf, 2021; Dummer et al., 2021). Treatment follows a stage-adapted approach, with skin-directed therapies in early stages, and systemic therapies in advanced disease (Kempf and Mitteldorf, 2021). CTCL is a chronic disease, with therapeutic goals focusing on symptom control, disease stabilization, and maintaining quality of life (QoL; Brunner et al., 2020; Roccuzzo et al., 2024; Bagot, 2025). Disease progression varies considerably between patients, but many live with their disease for years or decades, especially when diagnosed early (Agar et al., 2010; Scarisbrick et al., 2015). The 5-year overall survival rate ranges 78–96% in early-stage MF to 18–63% in advanced-stage disease, depending on risk stratification (Scarisbrick et al., 2025; Kersten et al., 2026). Most patients with MF are diagnosed at an early stage, predominantly at stage IA, with around 71% of patients presenting at stages IA-IIA (Benton et al., 2013; Maguire et al., 2020). Approximately 20–25% of early-stage patients progress to advanced disease (Scarisbrick et al., 2021; Kersten et al., 2026).

The symptoms associated with the disease, such as pruritus, pain and skin manifestation significantly impair patients’ QoL (Demierre et al., 2006; Beynon et al., 2015; Ottevanger et al., 2021; Bhat et al., 2021). This impairment is often stage-dependent, with advanced stages showing greater functional limitations, particularly disrupted sleep due to nocturnal pruritus and mobility limitations related to painful skin (Demierre et al., 2011; Herbosa et al., 2020; Ottevanger et al., 2021). Psychological distress including anxiety and depression commonly affects patients, especially women and those with advanced disease (Molloy et al., 2020; Ottevanger et al., 2021; Chalaka et al., 2023). Depressive feelings have been reported in 72.7% of CTCL patients (Demierre et al., 2006), and 30.7% of MF patients exhibit moderate-to-severe depression compared to 14.6% of healthy controls (Mohammadi et al., 2026). Patients also report social burden and work-related challenges due to visible skin lesions and treatment demands (Beynon et al., 2015; Ottevanger et al., 2021; Bhat et al., 2021). CTCL also creates financial burden through costs for topical treatment and frequent clinic visits, and affects family dynamics through caregiving needs (Selman et al., 2015; Bhat et al., 2021). Studies identified challenges in CTCL care, including diagnostic delays, communication issues, and access barriers (Scarisbrick et al., 2019; Bhat et al., 2021). In early-stage MF, the median time to a correct diagnosis is 3 years, with 85.6% of patients experiencing a delay in diagnosis (Scarisbrick et al., 2019).

While quantitative studies (Demierre et al., 2006; Wright et al., 2013; Molloy et al., 2020; Ottevanger et al., 2021) documented the general impact of the disease on patients’ QoL, only a few qualitative studies (Demierre et al., 2011; Beynon et al., 2015; Bhat et al., 2021) explored individual patients’ experiences in depth. However, existing QoL instruments fail to accurately capture disease-specific aspects such as sleep problems and concerns about appearance (Bhat et al., 2021; Gabes, 2021). Further research is needed to develop comprehensive CTCL-specific measurement tools (Jonak et al., 2019; Brazel et al., 2024). What remains also largely unexplored is the influence of care experiences on the psychosocial well-being of CTCL patients. Studies on other rare cancers show that challenges within the care system, such as diagnostic delays and a lack of expertise, often cause a significant psychosocial burden for patients (Keat et al., 2013; Low et al., 2024). It has also been reported that patients with rare cancers have unmet supportive care needs, such as information provision and psychological support (Heus et al., 2021). Psychosocial guidelines for patients with rare cancers in Germany are currently lacking.

To our knowledge, no research has examined how CTCL affects patients’ lives in Germany. Our study therefore aimed to gain deeper insights into the lived experiences of patients with this rare disease. We explored health-related quality of life (HRQoL), with a particular focus on psychosocial aspects. Additionally, our study examined how patients experienced the care pathway and what their care needs were.

## 2 Materials and methods

### 2.1 Study design

We conducted semi-structured interviews as part of the qualitative phase of a larger mixed-methods study. This exploratory approach aimed to generate new insights without predefined hypotheses. Ethical approval was obtained by the Ethics Committee of Westfalen-Lippe in Germany (reference: 2024-554-f-S).

### 2.2 Sampling and recruitment

Participants were recruited through a specialized multidisciplinary skin tumor center in North Rhine-Westphalia and two German CTCL support groups whose members were affiliated with specialized centers across Germany (9 centers total). Purposive sampling was used to achieve maximum variation in age, sex and disease stage. Patients with CTCL (MF and SS) aged 18 years or older and residing in Germany were included after written informed consent. We stopped recruitment when further interviews no longer provided new relevant insights, indicating thematic saturation. Due to the rarity of CTCL, the specificity of participants’ experiences ensured sufficient information power across the interviews (Malterud et al., 2016).

### 2.3 Data collection

Interviews took place between October 2024 and February 2025 via Zoom or telephone (voice only), based on participants’ preferences. We developed a semi-structured interview guide based on international literature. Central themes were systematically identified, grouped into main categories, and agreed upon within our research team. The guide included an open narrative prompt about disease history, allowing patients to share their experiences without predetermined questions. This was followed by thematic sections that systematically addressed physical, psychological, and social aspects according to the multidimensional understanding of HRQoL (Torrance, 1987; Ustjanauskas and Malcarne, 2020). We deliberately chose to focus on HRQoL rather than general QoL, as this sub-construct specifically examines disease-related impacts on functioning and well-being (Torrance, 1987; Ustjanauskas and Malcarne, 2020). Additionally, care experiences and needs were explored. This structure ensured comparability between interviews while allowing sufficient space for new participant topics. All interviews were conducted by a scientist experienced in qualitative research who had no prior contact with the participants, except for brief scheduling conversations. To reduce bias, our research team was not involved in clinical care, and the interviewer had no previous CTCL clinical experience to maintain analytical distance. Data collection continued until we reached consensus that no new themes emerged from the interviews.

### 2.4 Data analysis

All interviews were audio-recorded and transcribed verbatim, then checked for accuracy and pseudonymized. We conducted interviews in German and translated quotations accurately into English. Data analysis was performed using qualitative content analysis according to Kuckartz (Kuckartz, 2018) with MAXQDA software. After initial familiarization with the material, main categories were developed based on the research focus (HRQoL dimensions, care experiences/needs) and emerging themes from the data. The categories were inductively expanded and refined through iterative coding cycles, with subcodes developed to capture the full range of participants’ experiences. The coding framework was developed through regular discussions by the interdisciplinary research team (A. W., J. S., S. S.) and then consistently applied to all transcripts (A. W.).

To ensure analytical rigor, selected transcripts were independently coded by a co-author (J. S.), with coding decisions compared and discussed within our interdisciplinary team (two health scientists, one dermatologist). Discrepancies were resolved by consensus.

## 3 Results

Nineteen patients were interviewed (7 via Zoom, 12 by telephone). Five patients were recruited through the skin tumor center, 14 through support groups. The median interview duration was 47 min, totaling 955 min of audio recordings (range 19–76 min). Eleven women and eight men were interviewed, aged 35 to 80 years. Demographics resembled international CTCL populations (Dobos et al., 2020; Cai et al., 2022), though women were overrepresented in our sample. Table 1 shows the sample characteristics.

**Table 1:** Participant characteristics (*N* = 19).

Characteristics		*n*
Sex	Female	11
	Male	8
Age	< 45	3
	45–64	12
	≥ 65	4
Stage of CTCL	IA	4
	IB	4
	IIB	5
	III	2
	IV	4

### 3.1 Impact on health-related quality of life

The effects of CTCL on HRQoL varied among participants. While a few reported minimal or no impairment, the majority experienced limitations in certain situations or substantial impairments across HRQoL domains. Based on these, the following sections present the disease’s impact on physical, social and psychological well-being, as well as coping strategies. Table 2 provides supporting quotes from participants.

**Table 2:** Impact of CTCL on health-related quality of life.

Subtheme	Patient quote
Physical symptoms	*‘Yes, and the worst thing basically was this burning and this itching. And once you have started, you somehow can’t stop anymore.’ (I3, female)*
	*‘I had skin tears on my feet and on the inside of my hands. On my feet, so that it hurt so much that I could hardly walk.’ (I14, male)*
	*‘Actually, I only have these patches that don’t bother me either.’ (I10, female)*
Social impact	*‘My eyes hurt terribly because I’m so tired that they burn […] I find it very distressing that I then somehow drive to work crying in the morning […] People are wanted who are in a good mood. Who radiate optimism, who spread positivity. They are in demand. […] Those who are not resilient and not flexible are not so well regarded.’ (I5, female)*
	*‘I also notice that it affects my relationship, my marriage […] Everyone is afraid. Everyone has thoughts in their head. And that naturally leads to […] friction.’ (I10, female)*
Psychological impact	*‘I have days [...] where I feel totally good and then I have days where I don’t feel so good, then I just have to cry again because I ask myself: why me of all people?’ (I19, female)*
	*‘My psyche is not impaired in any way by this disease.’ (I2, male)*
	*‘We don’t get the diagnosis: you are healthy now. That doesn’t exist. And you don’t know when or how this disease will progress.’ (I11, female)*
	*‘That is like with every healthy person I think too. You have good days. There are days when the disease takes up a lot of space […] and then there are days when the disease […] has only such a very small place, but it is tiny. I believe it’s always a question of mindset.’ (I17, female)*
	*‘The only thing is that nobody actually knows what we are talking about, what we are affected by, nobody knows. Even a doctor doesn’t know. And it’s really difficult to deal with it again and again.’ (I5, female)*

#### 3.1.1 Physical symptoms and impact on daily life

The most frequently reported symptoms were pruritus, skin redness, and flaky skin. Pruritus, which often intensified at night, caused considerable sleep disturbances and exhaustion that impaired several patients’ performance in both work and daily activities:

*‘I sleep poorly, I am exhausted. I have itching so that I get up all night and get cool packs and put them on my skin. I am done.’ (I8, female)*

Additionally, patients experienced pain, skin fissures, and open, weeping lesions. Some patients presented with additional symptoms such as nodules, an increased susceptibility to infection, fever episodes, weight loss, and fluid retention. One patient described it as follows:

*‘The worst part is, when so and so many percent of the skin are covered, fever spikes occur and they are extreme for me […] you think the world is ending.’ (I17, female)*

Beyond disease-related symptoms, several participants reported treatment-related side effects, such as hair loss, concentration difficulties, neuropathy, skin thinning (‘parchment skin’), and loss of appetite. Patients in advanced disease stages often experienced a greater physical symptom burden, though physical symptoms caused burden across all stages. However, several participants also reported significant improvements in symptoms through treatments and, consequently, improved HRQoL.

#### 3.1.2 Social impact

Visible skin changes influenced patient behavior, with participants reconsidering public activities:

*‘There are days when it is very visible […] where I think very consciously in the morning about whether I want to go out in public with it or not.’ (I1, male)*

Consequently, some patients tried to hide affected skin areas with long-sleeved clothing or make-up. Several participants reported stigmatizing experiences in public, which resulted in reduced social contact and leisure activities. Some patients avoided crowds due to fear of infection. Moreover, fatigue reduced participants’ energy for social activities. On occasion, the disease also had a negative impact on relationships with friends and partners, which participants attributed to the emotional burden.

In their personal environment, patients primarily received encouragement and support from family members and friends, which proved to be an important resource for coping with the disease. However, in professional contexts, some experienced dismissive attitudes and a lack of understanding. As one patient explained:

*‘My former boss immediately said that it wasn’t that bad. That it wasn’t real cancer.’ (I8, female)*

However, occasionally, participants reported being supported by their employers, for example through the option of working from home. The time required for treatment also restricted several patients’ social and work lives. Appointment coordination, treatment sessions, and long-distance travel to specialized centers reduced the time available for other activities.

#### 3.1.3 Psychological impact

The most common psychological burden was caused by prognostic concerns, such as worries about disease progression, the risk of later metastasis and reduced life expectancy:

*‘I was always a bit scared and couldn’t look to the future with much hope.’ (I13, male)*

The unpredictable course of the disease resulted in feelings of lost control in several patients. Moreover, visible skin changes affected how many participants perceived themselves, which caused feelings of shame and diminished self-esteem:

*‘And I also had the feeling, how should I say it, that I’m no longer worth anything as a woman, because I’m simply no longer the person I used to be, in terms of my hair and my whole appearance.’ (I19, female)*

Some participants reported depressive symptoms and emotional exhaustion. Treatments created additional psychological distress for some patients. Factors that contributed to this burden included organizational challenges, complex medication administration, and the need to adapt daily routines:

*‘There was only the disease left, and I no longer existed. I as a person no longer existed.’ (I11, female)*

Additional occupational and financial concerns also burdened several participants. Balancing treatment demands such as the necessity for frequent appointments with work responsibilities was particularly challenging:

*‘So what scares me the most right now is worrying about my job. How can I manage that? Going to radiotherapy four times a week and still going back to work […] it’s all connected with financial restrictions.’ (I10, female)*

Some patients were concerned about losing their jobs. Many applied for disability certification, which offered some employment protection. However, they described the application process as psychologically challenging. Recognition was often difficult as the disease is relatively unknown, with its impact often dismissed by others as ‘just skin problems’. Some also struggled with the cost of self-funded skincare products. Overall, patients at both early and advanced stages experienced psychological burden.

#### 3.1.4 Coping with the disease

Patients developed various coping strategies to manage their disease. Social support from family, partners, and friends emerged as a central resource. Support groups were an important source not only of information exchange, but also of emotional support for several participants:

*‘And the exchange was for me like, yes, well, it brought life back to me. That was wonderful. Really wonderful.’ (I8, female)*

Additional strategies used by participants included exercise, psychological support, meditation, reading, research, travel, work and interaction with pets. Some participants developed a deliberately positive attitude, while others consciously avoided thinking about their disease.

### 3.2 Care experiences and needs

While approximately half of the patients expressed satisfaction with their current care, the other half reported dissatisfaction and identified areas for improvement. Most currently satisfied patients had previously faced care challenges that were resolved over time, such as finding a specialized doctor after multiple consultations. The following sections present themes considered important by patients: treatment pathways, communication and information needs, and systemic challenges. Table 3 illustrates care experiences, while Table 4 presents wishes for improvements.

**Table 3:** Care experiences.

Subtheme	Patient quote
Access to care	*‘No dermatologist wanted to take me because of the disease. I called 14 doctors. One said quite nicely: “I would take you, but your disease is simply too expensive for a doctor in private practice.”’ (I17, female)*
Diagnostic process	*‘In retrospect, it was a disaster with the first doctors up to the clinic, because nobody even thought about it. Me neither, of course. And they did all sorts of things. So it was trial and error.’ (I7, male)*
	*‘But they were not sure exactly what it was and how severe it was. […] It was still a matter of: I’ll fall over the next day or it’s just mild, severe neurodermatitis and the whole thing dragged on for months and weeks. Of course, that does a lot to you.’ (I1, male)*
Diagnosis communication	*‘The doctor doesn’t look at me, no explanation, nothing. And what’s stuck with me? I have cancer. I have to die […] And then, of course, I started to cry. Then she was completely overwhelmed.’ (I10, female)*
	*‘I was told the diagnosis over the phone, which I found extremely tasteless.’ (I17, female)*
Access to information	*‘I wasn’t provided with any information. What I would have liked was a flyer from a support group when I was in a clinic for the first time.’ (I8, female)*
	*‘But unfortunately, it’s just the way it is today in the medical profession in general that you go there, get a diagnosis, get medication and then you are allowed to leave. Unfortunately, that’s the way it is all the time and you always have to do your own research.’ (I16, male)*
	*‘I find very, very little information about the disease itself. And I have to say, the clinic, the doctor really supports me there or provides me with a lot.’ (I6, male)*
Doctor-patient communication	*‘I just wish I could get in there and not be asked about my skin first, but simply asked: How are you? Are you feeling well?’ (I5, female)*
	*‘We can ask any question we have there. And if we ask it five times, that’s okay too.’ (I1, male)*
	*‘And the problem is, if you want to know more or learn more, then you have to pay for the session privately.’ (I19, female)*
	*‘But there is a roadmap [for other types of cancer], every doctor knows what to do. No doctor would think of saying: “Oh, it’s not so bad,” because I hear that constantly […] I always have the feeling that I have to go into defensive mode, because I don’t die immediately, but later.’ (I17, female)*
Continuity of care	*‘There’s always a different assistant doctor. I’ve already had four or five different assistant doctors. They know what it’s about when they look at the computer, and they look at me and prescribe the tablets.’ (I19, female)*
Psycho-oncological support	*‘But just try finding a psychologist when you actually need one.’ (I4, male)*
	*‘There are always intervals where you can use it [psychological support] and then it expires. And with an illness like that […] I’ll just say it like this: it just sucks.’ (I5, female)*
	*‘And then I immediately got a therapy appointment with a psychologist there at the clinic […] That helped me.’ (I8, female)*

**Table 4:** Patient wishes for improved care.

Subtheme	Patient quote
Information access	*‘Well, the [ideal care] would look like me being guided through it. That means it wouldn’t only rely on my own initiative, but rather that when I get such a diagnosis, they would say: okay let’s have a half-hour conversation with me and then explain what it means, what effects it has, medications, what options there are for it. Simply these pieces of information that I have now collected afterwards, that I would actually get them there automatically, that would actually be the goal.’ (I16, male)*
	*‘I wish there was a bit more information available to those patients who want it.’ (I6, male)*
	*‘I would love to have a checklist. I want an empathetic doctor who teaches me as gently as possible what disease I have, and then, in the same breath, gives me an information brochure with all the information […]: Today you were diagnosed with MF. Please take care of the following next […] If these symptoms occur, then you react like this. So that you really have a handbook about MF […] like a washing machine manual that you can look into. Error 14 - What do I do? That would be great.’ (I10, female)*
	*‘Yes, for the patients who are interested, that they really have a chance to access scientific articles, perhaps also in cooperation with the skin center that is treating them. That there is an opportunity for them to offer such access […] and yes, that is a very, very great wish.’ (I1, male)*
Doctor-patient communication	*‘But it would be very, very, very helpful for patients if the medical communication is designed in such a way that on the one hand the patient understands it and on the other hand you come there and don’t have the feeling you are one of many, because that’s just not what you are. With this illness you just aren’t that.’ (I15, female)*
	*‘My dream would simply be to find a dermatologist who is familiar with the disease and is willing to treat me and maybe even say: “Hey, we have a new treatment method.” My dermatologist says: “I have no idea. If you read anything about it, you can tell me.”’ (I17, female)*
	*‘[…] maybe with young doctors this could be a bigger point in the future: how do I deal with patients at all?’ (I11, female)*
Psycho-oncological support	*‘What is also important to me is […] for the moments when it might get a bit dark, that I think I could get psychological support more easily than is possible nowadays. That would be really important to me, if that were possible, that I think, I’m having such a low point right now. I would need someone who would gladly help me. A professional.’ (I4, male)*
Care coordination and continuity	*‘So the ideal care would basically be […] a well-structured and communicatively well-built structure, so connection to the clinic. So this just continuity in the form of the doctors. Nobody says it has to always be one doctor, but maybe just two.’ (I15, female)*
	*‘The most important thing is […] why don’t dermatologists create a network, where they all write down: I have this patient, who has this and that, and I treated it with this, and this had positive or negative effects, where every dermatologist could look it up.’ (I14, male)*
Social acceptance	*‘That would also be a wish for the future, that this topic of cancer becomes destigmatized […] yes, people can talk about it today when they have psoriasis, so why shouldn’t they be able to talk about it when they have cancer.’ (I1, male)*

#### 3.2.1 Treatment pathway

Finding an appropriate dermatologist posed an initial barrier for most patients, often with long waiting times. Several participants noted substantial differences in appointment availability depending on their insurance status. Limited CTCL expertise among non-specialized doctors and considerable distances to specialized centers hindered access to adequate care:

*‘This happens to almost all of us: we have skin problems and in many cases it is incorrectly assessed, diagnosed and treated. That’s how it was for me too. […] I went to a handful of dermatologists and each one said something different.’ (I14, male)*

Several patients reported that seeking specialist care required substantial personal initiative. Many experienced prolonged diagnostic pathways with misdiagnoses or only initial symptomatic treatment, as the condition was frequently mistaken for more common skin conditions such as eczema or psoriasis. The diagnostic process often extended over years. Even after having found a dermatologist, the diagnostic process still remained complex.

*‘I have to say, I was very fortunate with my treatment. I could have ended up somewhere completely different.’ (I1, male)*

This statement reflects the experiences of several patients who indicated that chance or fortune played an important role in their diagnostic journey. One participant who had previously survived a more common cancer highlighted the absence of clear pathways. She noted that care for her previous cancer followed established protocols, like a ‘roadmap’, whereas CTCL lacked such structures.

Few patients reported positive experiences in which doctors immediately initiated biopsy or blood investigations at the first consultation, which accelerated the diagnostic process:

*‘The doctor suspected I had neurodermatitis, but the female doctor really wanted a biopsy to be done. On the fourth or fifth day, the doctor calls me and says: “come down, we now have the result.”’ (I19, female)*

Furthermore, many patients experienced changing doctors throughout their care and found the lack of continuity burdensome, resulting in a fragmented treatment process. This forced patients to repeatedly recount their medical history during follow-up appointments. Consequently, this shortened the consultation time and limited time for questions, which impaired the quality of the communication. Inadequate information transfer within the care system further complicated continuity of care:

*‘But what really surprised me a lot. I have now had my patient file set up for me […] and there I discovered that this MF had already been diagnosed by my previous dermatologist. Yes, but I didn’t know anything about it.’ (I16, male)*

Patients also criticized the lack of interdisciplinary collaboration between different medical specialties. Many desired a more holistic approach to their disease, for example through involvement of psycho-oncology services. Some patients also reported that they had to organize contact with specialized centers themselves without support. However, a few patients also had positive experiences with stable, trust-based doctor-patient relationships, which they found very helpful:

*‘With my doctor, it’s not like with specialists or at the hospital: no time, no time, no time. Instead, they take the time.’ (I1, male)*

#### 3.2.2 Communication and information needs

The diagnostic consultation was a challenge for many patients, not only because of the diagnosis itself, but also because doctors provided inadequate information and showed little empathy. Patients often received minimal or no information about the disease, its progression, or available treatment options. This frequently resulted in substantial unmet information needs and patients reported to feel abandoned:

*‘I looked at the doctor and said: how long do I have left? Do I need to start writing my bucket list? […] I was completely overwhelmed. She got overwhelmed too and abruptly left the room.’ (I11, female)*

Many patients consequently sought information online independently, which sometimes caused anxiety due to distressing content. Patients valued support groups as reliable information sources. However, some patients did report satisfactory doctor-patient communication and adequate information provision:

*‘I ended up with a really great doctor. Fortunately […] and he explained it to me really well.’ (I4, male)*

#### 3.2.3 Lack of recognition and systemic barriers

Within healthcare settings, several participants frequently felt they were not taken seriously and that their condition was trivialized, which created additional burden:

*‘I have the feeling that because it’s only the skin, it’s a second-class cancer.’ (I17, female)*

Many patients also described feeling unheard and not properly understood:

*‘I don’t feel taken seriously. […] I simply don’t feel understood enough. But I think several others feel the same way.’ (I19, female)*

Often, patients had to justify themselves and prove the severity of their disease:

*‘But this way doctors handle the disease, it’s really unpleasant. It really makes things difficult for you. You always feel like you’re a supplicant.’ (I17, female)*

Two participants who had survived a more common cancer type described differences in how their disease was recognized. While their previous cancer was immediately recognized as serious, CTCL required justification because physicians often dismissed it as ‘not that bad’. One participant stated she would rather have her previous cancer again than CTCL. She experienced CTCL as more life-limiting than her previous cancer, yet physicians did not acknowledge this. The constant need to justify her condition was particularly a psychological burden.

Similarly, patients faced challenges when applying for disability certification, as authorities often hardly understood CTCL and trivialized the disease. Many participants reported rejection of their applications and repeated requests for documentation:

*‘It’s a total battle with the authorities because they also have no idea what this is. […] Unfortunately, you don’t get permanent disability certification, but only for three or five years. Last time they wanted to revoke it again.’ (I7, male)*

Numerous patients reported that this process was mentally exhausting and emotionally distressing. Patients also perceived regional disparities because certain regions applied more stringent practices.

Furthermore, patients experienced problems accessing psycho-oncological support due to insufficient information from doctors and systemic barriers from health insurance companies, which often provided a limited number of sessions. Once these were used, patients had to wait again for approval, a process they found very burdensome:

*‘But there are also phases when I really need her [psychologist]. […] For me, it’s already a horrible thought that she will be taken away from me at some point.’ (I11, female)*

## 4 Discussion

This study is the first to explore HRQoL, care experiences and needs of patients diagnosed with CTCL within the German population. The chronic disease has been known to persist for extended periods, often spanning years or even decades, particularly in cases where it is diagnosed in its early stages. Consequently, the need for ongoing care becomes a central aspect of the patient’s life. It is crucial to comprehend patients’ experiences of care, their unmet needs, and the impact of the disease on their daily lives in order to develop patient-centered care models and enhance the quality of care.

The present findings are in alignment with those of international research, which demonstrates that QoL among patients suffering from CTCL is impaired across the physical, psychological and social domains (Demierre et al., 2006; Beynon et al., 2015; Molloy et al., 2020; Ottevanger et al., 2021; Bhat et al., 2021). These domains were found to be interconnected, with physical symptoms contributing to the patient’s psychosocial burden. For instance, the presence of visible skin changes has been shown to result in both external stigmatization and internalized stigma, as well as social withdrawal. However, while earlier research has indicated an association between advancing disease stage and reduced QoL (Herbosa et al., 2020; Ottevanger et al., 2021), our study reveals a more nuanced perspective. Patients in the early stages of the disease were also severely affected, but with different primary concerns. While physical symptoms were less evident, psychological burden frequently emerged due to unmet care needs. Care-related burden was also reported at more advanced stages.

These findings underscore the pivotal function of comprehensive, interdisciplinary care, as favorable experiences involving continuous, empathetic support exerted a positive influence, while adverse care encounters frequently resulted in a substantial augmentation of psychological distress. As demonstrated in the research, care experiences have been shown to have a significant impact on the well-being of cancer patients (Drury et al., 2024). Furthermore, the continuity of care has been identified as a crucial factor in determining patient outcomes in chronic conditions (van Servellen et al., 2006). The present study further supports these findings and additionally identifies specific care requirements for patients with CTCL that have not been systematically examined.

A clear illustration of this are the challenges encountered during the diagnostic process. The participants reported prolonged and fragmented diagnostic journeys, a finding that is consistent with the results of previous research (Scarisbrick et al., 2019; Boh et al., 2023). This is likely due to the fact that early stages often resemble benign inflammatory skin conditions, such as eczema or psoriasis, which can result in diagnostic delays (Litvinov et al., 2017). The findings of this study demonstrate that protracted diagnostic journeys and restricted information availability result in considerable patient distress. A paucity of information was a commonly reported unmet need, with patients emphasizing that understanding their disease was often essential to cope with their condition.

This highlights the importance of providing comprehensive, empathetic patient education at the time of diagnosis, supported by targeted informational materials on the disease, treatments, and support services. It is equally important to enhance awareness of CTCL among doctors, which will improve their ability to recognize symptoms and shorten diagnostic pathways. This necessitates the systematic incorporation of CTCL-specific content into medical education, specialist training, and continuing professional development, as outlined in national and international action plans for rare diseases [Nationales Aktionsbündnis für Menschen mit Seltenen Erkrankungen (NAMSE), 2013; Tumiene et al., 2022].

In addition to medical education, it is imperative to prioritize raising public awareness of CTCL. It was evident that patients encountered challenges in a variety of settings, including healthcare, occupational environments, and administrative procedures, largely attributable to their limited awareness. Several patients were required to provide substantiation for the validity of their disease and symptoms. This was due to the fact that CTCL was frequently disregarded as ‘merely a form of skin cancer’ or alternatively characterized as a ‘second-class cancer’. This trivialization is at odds with extant literature and the findings of the present study, which demonstrate that both visible and invisible symptoms can substantially reduce patients’ QoL (Demierre et al., 2006; Beynon et al., 2015; Ottevanger et al., 2021; Bhat et al., 2021). The minimization of the impact creates an additional psychological burden. It is therefore essential to enhance awareness among pivotal stakeholders, a group which encompasses medical assessors who are responsible for the determination of disability recognition decisions. Beyond healthcare settings, it is important to raise awareness among the general public to improve societal understanding of rare diseases and the challenges faced by patients.

Despite the limited recognition of this phenomenon, many patients experienced severe psychological burden, including symptoms of anxiety and depression. Psychological distress was evident at all stages of the disease, yet its severity exhibited variability among patients, potentially indicative of differences in individual coping mechanisms and care experiences. As patients developed various strategies to manage their disease and its associated burden, factors such as social support from family and friends, or information exchange within support groups, may help to mitigate psychological distress. It is noteworthy that the psychological burden appears to originate not only from the disease itself, but also from its rarity. Challenges such as diagnostic delays, lack of recognition, and limited specialist expertise created substantial distress. These findings are consistent with those from research on other rare cancers, which also documents psychosocial burdens linked to challenges in care (Keat et al., 2013; Low et al., 2024).

However, only a small proportion of patients received adequate psychological support, although those who accessed such services reported positive outcomes. Many patients reported that treatment focused too narrowly on physical symptoms. Consequently, psychological support should be systematically integrated into patient care. It is recommended that regular screening during follow-up appointments be carried out using validated instruments such as the Distress Thermometer, with a view to facilitating early identification of psychological distress. The efficacy of this instrument in screening has been demonstrated (Mitchell, 2007), and its implementation in a German skin cancer center has yielded positive outcomes (Wünsch et al., 2024). Furthermore, it is essential that medical practitioners provide information regarding psychological support services, irrespective of the disease stage. To enable physicians to make effective referrals and guide patients toward appropriate support, training in the recognition of psychosocial distress is essential. Specialized centers should offer psychological services or establish regional collaborations to provide such support [Nationales Aktionsbündnis für Menschen mit Seltenen Erkrankungen (NAMSE), 2013]. Additionally, it is recommended that psycho-oncologists receive training in the management of rare diseases.

Nevertheless, in contrast to the comprehensive and disease-specific psychological care recommendations outlined in other German cancer guidelines, the CTCL guideline (Dippel et al., 2022) predominantly refers to general psycho-oncology guidelines. Furthermore, psycho-oncological support is often subject to time limits imposed by health insurers, which may not adequately address the chronic nature of CTCL. Patients reported that interruptions in psychological care due to session limits were particularly burdensome. These interruptions create care gaps that could be prevented by more appropriate reimbursement structures. This may reflect a broader structural gap in policies affecting many patients with chronic diseases who are seeking ongoing psychological support. For patients with rare diseases such as CTCL, this challenge may be further compounded by a lack of awareness of the condition’s severity and its chronic impact. Therefore, raising awareness among policymakers and insurers is crucial, as this could help facilitate flexible approval models tailored to patients’ needs, for example through extended reimbursement periods. Given the psychological challenges identified in the present study, the inclusion of more specific recommendations for psychological care in the guidelines for CTCL would represent a significant step toward the provision of more holistic patient care.

In addition to difficulties accessing psychological support, patients reported challenges with care coordination and continuity. These challenges may be indicative of more extensive systemic issues in the management of rare diseases, where expertise is frequently distributed and restricted (Stoller, 2018). This issue is evident in Germany, where patients with rare diseases encounter a healthcare system that is deficient in the provision of coordinated specialized care (Schlangen and Heuing, 2023).

One potential strategy to address this issue is to concentrate expertise in a limited number of highly specialized lymphoma centers with multidisciplinary teams, including specialists in dermatopathology, molecular diagnostics, and psycho-oncology. It is recommended that such centers establish collaborative relationships with outpatient dermatologists and general practitioners, and that they designate clear contact persons for communication. This approach is analogous to the hub-and-spoke model, in which hubs (expert centers) collaborate with spokes (outpatient physicians) via structured referral pathways (Elrod and Fortenberry, 2017). Research has demonstrated the efficacy of this approach in the context of rare diseases. The findings indicate that it can enhance diagnostic accuracy, the quality of treatment, and patients’ access to care (Elrod and Fortenberry, 2017; Zucchetti et al., 2018; Tosi et al., 2020; Lanzkron et al., 2024). Furthermore, these centers have the capacity to provide targeted physician training and implement guideline-based management pathways.

Despite these challenges, the study also uncovers notable strengths in the current care that could inform future enhancements. Several patients reported high satisfaction with their care, attributing this to factors such as effective communication with doctors, the provision of adequate information, and the establishment of long-term care relationships. Furthermore, patients particularly valued the coordination of transitions between medical specialties. This finding is consistent with the findings of research on continuity of care in chronic diseases, which identifies sustained doctor-patient relationships, coordination, and information transfer as key attributes that improve quality of life and patient satisfaction (van Servellen et al., 2006; Hu et al., 2020; Wang et al., 2025). Moreover, a project centered on rare diseases in Germany demonstrated that a coordinated collaboration between centers, interdisciplinary case conferences, and interregional case management can enhance diagnostic accuracy and treatment quality (Schlangen and Heuing, 2023). These experiences and examples underscore the potential for enhancing the quality of care.

The study’s limitations can be attributed to two factors: firstly, the sample size was relatively small; and secondly, the study was conducted within a German healthcare context, which restricts the generalizability of the findings. The slightly higher proportion of female participants should be considered when interpreting the study findings, given gender-specific HRQoL differences (Chalaka et al., 2023). Consequently, this gender imbalance may have led to a higher reported psychosocial burden than would be observed in a more gender-balanced sample. The collection of demographic data was limited, which precludes conclusions regarding socioeconomic and cultural diversity.

Strengths of our study include being the first to examine care experiences, needs and HRQoL among CTCL patients in Germany. Data saturation was reached with 19 interviews that covered various disease stages and ages. The exploratory approach facilitated the acquisition of new insights that would not have been captured through standardized instruments. Moreover, one-to-one interviews conducted by an independent researcher helped mitigate potential biases and facilitated an open dialog. Multiple recruitment pathways provided a comprehensive perspective on nationwide care.

In conclusion, the majority of patients in both the early and advanced stages experienced a disease-related burden across the physical, psychological, and social domains, albeit with varying patterns. A salient finding of the study is the pivotal role of the quality of care in influencing patients’ HRQoL. The findings of this study demonstrate that the psychosocial burden experienced by patients is not solely attributable to the nature of the disease itself, but is also considerably influenced by its rarity and the subsequent challenges associated with the delivery of care. The provision of adequate care has been demonstrated to enhance psychosocial well-being and facilitate patients’ coping mechanisms. Conversely, the absence of met care needs can impose an additional burden. Despite the evidence that care experiences can influence patient outcomes in chronic conditions, the perspectives of CTCL patients on their care have only been addressed in a marginal way. This study provides the first systematic examination of care experiences and their impact on the psychosocial well-being of patients with CTCL, with important implications for the development of HRQoL instruments that are better able to capture patient experiences.

These results emphasize the necessity for a multifaceted care strategy, encompassing integrated psychological support, structured patient education, and coordinated care pathways. Given the rarity of CTCL, there is a necessity for specialized centers to be equipped with multidisciplinary teams, thus facilitating the systematic addressing of identified care gaps. The utilization of positive care experiences as benchmarks for improvement serves as a crucial component in this process.

The findings of the present study provide a foundation for future research and approaches to optimize care delivery. A cross-sectional study with a larger sample is planned to validate these findings.

## Data Availability

The datasets for this article are not publicly available due to concerns regarding patient anonymity and ethical restrictions. Requests to access the datasets should be directed to the corresponding author.
